# Multi-Cohort High-Dimensional Proteomics Reveals Early Risk Markers for Lymphoid Cancer Subtypes

**DOI:** 10.21203/rs.3.rs-7400676/v1

**Published:** 2025-08-19

**Authors:** P. Martijn Kolijn, Karl Smith-Byrne, Vernon Burk, Vivian Viallon, Matthew A. Lee, Keren Papier, Ziqiao Wang, Anton W. Langerak, Florentin Späth, Arjan Diepstra, Christina M. Lill, Raul Zamora-Ros, Alessandra Macciotta, Amaia Aizpurua, Rosario Tumino, Nilanjan Chatterjee, Ruth C. Travis, Marc J. Gunter, Elizabeth A. Platz, Elio Riboli, James McKay, Roel C.H. Vermeulen

**Affiliations:** Utrecht University; University of Oxford; Johns Hopkins Bloomberg School of Public Health; International Agency for Research on Cancer (IARC) - World Health Organization; International Agency for Research on Cancer (IARC) - World Health Organization; University of Oxford; Johns Hopkins Bloomberg School of Public Health; Erasmus MC; Umeå University; University of Groningen; University of Münster; Bellvitge Biomedical Research Institute (IDIBELL); University of Turin; Biodonostia Health Research Institute; 17. Hyblean Association for Epidemiology Research; Johns Hopkins Bloomberg School of Public Health; University of Oxford; Imperial College London; Johns Hopkins Bloomberg School of Public Health; Imperial College London; International Agency for Research on Cancer (IARC) - World Health Organization; Utrecht University

**Keywords:** Lymphoma, Multiple Myeloma, Proteomics, Early detection

## Abstract

This study aims to investigate the early stages of lymphoid malignancy pathogenesis and identify novel pre-diagnostic proteomic markers for lymphoma. Using the SomaScan-7K platform, we analyzed 6,412 unique plasma proteins in a case-cohort study nested within the European Prospective Investigation into Cancer and Nutrition (EPIC) cohort, comprising 4,565 participants (484 incident lymphoid malignancy cases, median follow-up 9 years). We identified over 500 unique protein-lymphoid malignancy associations. Enriched pathways included viral protein interactions, cytokine signaling, B-cell receptor signaling, and NF-κB activation, reflecting key mechanisms in lymphoma pathogenesis. Cross-cohort validation of the top 20 FDR-significant proteins revealed concordant nominal significance for 70%−95% of the associations in the UK Biobank (Olink) and ARIC (SomaScan) studies. Time-stratified analyses revealed that a subset of these protein-lymphoma associations is evident over a decade before diagnosis. These findings highlight the potential of circulating proteomic markers in risk stratification, early diagnosis, and targeted prevention strategies for lymphoid malignancies.

## Introduction

Lymphoid malignancies comprise a heterogeneous group of cancers varying in etiology, incidence, and survival.^1–8^ Notably, several lymphoid malignancy subtypes are preceded by precursor conditions detectable years before overt malignancy. Examples of precursor conditions include: monoclonal B cell lymphocytosis (MBL) preceding chronic lymphocytic leukemia (CLL), circulating t(14;18)-positive B-cells preceding follicular lymphoma (FL), non-IgM isotype monoclonal gammopathy of undetermined significance (MGUS) preceding multiple myeloma (MM,) and IgM isotype MGUS preceding Waldenström macroglobulinemia.^9–13^ Studying the early development of lymphoid neoplasms holds the potential to reveal key insights into the etiology of these precursor conditions and the biological drivers contributing to progression to overt malignancy.

Circulating proteins play an essential role in both the early pathogenesis of lymphoid malignancies and the immunological response to these neoplasms.^14,15^ Proteomic studies have identified prediagnostic changes in cytokines and B-cell activation markers, with longitudinal analyses confirming alterations in markers such as sCD23 (FCER2), sCD27, sCD30, and CXCL13 up to two decades before lymphoma diagnosis.^16–28^ Several large studies, including the UK Biobank and Uppsala-Umeå Comprehensive Cancer Consortium (U-CAN), have employed high-throughput proteomics, analyzing panels ranging from 1,463 to 2,963 proteins.^29,30^ These studies identified hundreds of novel protein-cancer associations and reproduced established lymphoma-protein associations, such as soluble B-cell maturation antigen (sBCMA) with MM, sCD23 with CLL and CXCL13 with diffuse large B-cell lymphoma (DLBCL). However, these studies were constrained by the relatively limited number of proteins assessed (1,463–2,963) and the absence of cross-cohort validation across different proteomic platforms.

To address these limitations, we conducted high-throughput proteomic profiling of 6,412 circulating proteins using the SomaScan 7K Assay^®^ in a case-cohort study nested within the European Prospective Investigation into Cancer and Nutrition (EPIC) cohort. The study included 4,565 participants, 484 of whom developed lymphoid malignancies, with plasma samples collected a median of nine years before diagnosis (range 0.1–19 years). We performed validation analyses in the UK Biobank (Olink platform) and the Atherosclerosis Risk in Communities (ARIC) study (SomaScan-5K assay). This study aims to deepen our understanding of the plasma proteome in the early pathogenesis of lymphoid malignancies and identify novel markers for future risk prediction.

## Results

Baseline characteristics of the EPIC participants are shown in Table 1. The 484 incident lymphoid malignancy cases were diagnosed a median of 9 years (range 0.1–19 years) after blood collection. Compared to the sub-cohort, participants who later developed lymphoid malignancy were on average older and more likely to be male, consistent with previous reports (Table 1).^31^

### Proteomic Associations with B-cell lymphoma

**Figure 1:**
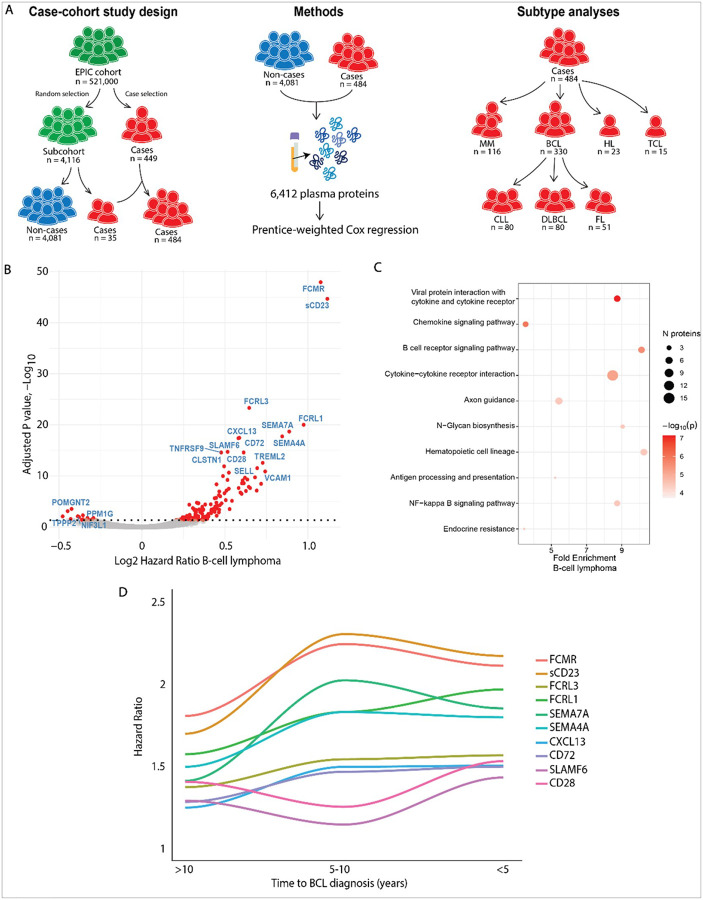
Protein associations with B-cell lymphoma. A) Graphical abstract of the study describing the study design, experimental approach and subtype analyses. For further details on the subtype grouping see **Supplementary Table 1**. B) Volcano plot of the hazards ratio and FDR-adjusted P-value as determined through Prentice-weighted Cox regression models for the risk of BCL for all aptamers included in the study (330 BCL cases and 4,088 non-cases were included in this analysis). C) Pathway enrichment analysis results for all 157 FDR-significant hits for BCL, top 10 pathways ranked by P-value are shown. For further details on the pathway analysis results, see **Supplementary Table 4**. D) Trajectory analysis of the hazard ratio of the top 10 proteins ranked by FDR-adjusted p-value. Cases were divided in 3 bins (151 BCL cases diagnosed over 10 years after blood collection, 106 BCL cases diagnosed between 10 to 5 years of blood collection, 73 BCL cases diagnosed within 5 years of blood collection), each bin was compared to 4,088 non-cases using Prentice-weighted Cox regression. For further details on the time-stratified analyses for BCL, see **Supplementary Figure 3**.

In our fully-adjusted models for B-cell lymphoma (BCL, n = 330), we observed 157 unique protein-BCL associations (173 aptamers), of which 139 were associated with an increased risk and 18 were associated with a decreased risk of BCL (Fig. 1B, **Supplementary Table 2**). BCL development was associated with increased plasma levels of members of several major immunomodulatory protein families, including the FC-receptor family, the semaphorin family, the TNF-receptor and TNF-ligand superfamily, the leukocyte immunoglobulin-like receptor family, the interleukins and a selection of chemokines promoting migration of activated lymphocytes (Fig. 1B, **Supplementary Table 2**). Pathway enrichment analysis for the 157 proteins associated with BCL revealed an enrichment of proteins associated with processes such as cytokine and chemokine signaling, B-cell receptor (BCR) signaling, the NF-κB signaling pathway, hematopoietic cell lineage, antigen processing and presentation and Nglycan biosynthesis (Fig. 1C, **Supplementary Table 4**).

### Time-stratified analyses for B-cell lymphoma

In time-stratified analyses of 151 BCL cases with an early blood sample who were diagnosed more than 10 years after blood draw, we observed 20 significant protein-BCL associations (**Supplementary Fig. 3A, Supplementary Table 3**). Early markers for BCL development included proteins associated with BCR signaling and the Fc receptor family such as FCMR and sCD23. For 106 BCL cases with blood samples collected between 5 to 10 years before diagnosis, we also observed 29 significant protein-BCL associations (**Supplementary Fig. 3B, Supplementary Table 3**). The top associated proteins included CXCL13, FDCSP, SELL and several members of the Fc-receptor and semaphorin families. For the 73 BCL cases with blood samples collected less than 5 years prior to diagnosis, we observed 201 significant protein-BCL associations (**Supplementary Fig. 3C, Supplementary Table 3**). Pathway analysis revealed an enrichment of the cytokine, chemokine, BCR and NF-κB signaling pathways (**Supplementary Fig. 3D, Supplementary Table 4**). In total, nine proteins showed consistent associations with BCL across all time intervals from > 10 years to < 5 years before diagnosis (**Supplementary Fig. 3E**). Trajectory analyses for the top 10 proteins associated with BCL ranked by FDR-adjusted P-value revealed increasing hazard ratios for FCMR, sCD23, FCRL1, FCRL3, CXCL13, CD72 SEMA7A and SEMA4A in blood samples drawn within 5 to 10 years before diagnosis, compared to participants with blood samples drawn over 10 years before diagnosis (Fig. 1D). The hazard ratio’s for FCMR, sCD23, FCRL3, CXCL13, CD72, SEMA7A and SEMA4A then reached a plateau, while the hazard ratio for FCRL1 continued to increase linearly among individuals with a blood draw within 5 years of BCL diagnosis. Hazard ratio’s for SLAMF6 and CD28 remained relatively stable across all time intervals from > 10 years to < 5 years before diagnosis.

### B-cell malignancy subtype specific analyses for CLL, DLBCL, FL and MM

**Figure 2:**
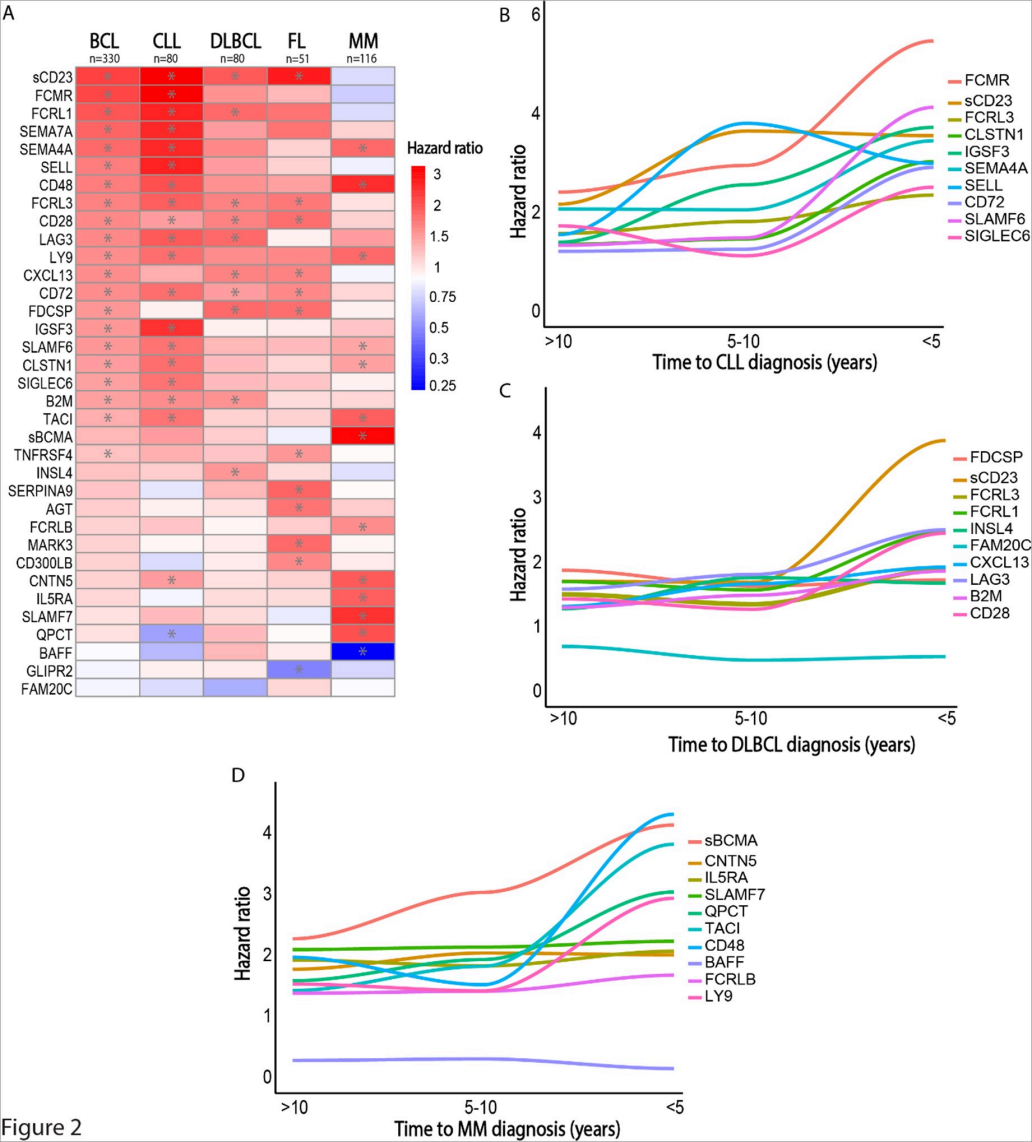
B-cell malignancy subtype analyses. A) In this heatmap, each row represents the hazard ratio for a protein resulting from Prentice-weighted Cox regression for the outcome listed for each column. FDR-significant associations are marked with a star. Only the top 10 associated proteins sorted by p-value are shown for each comparison. For each column, the number of lymphoid malignancy cases listed at the top of the column was compared to the full subcohort (N=4,116) through Prentice-weighted Cox regression. Color intensities are based on a log2 transformed scale of the hazard ratio (with blue representing a hazard ratio below 1 and red representing a hazard ratio above 1). Untransformed hazard ratio values are shown in the legend to facilitate interpretation. B) Trajectory analysis of the hazard ratio of the top 10 proteins associated with CLL ranked by FDR-adjusted p-value. Cases were divided in 3 bins (31 CLL cases diagnosed over 10 years after blood collection, 29 CLL cases diagnosed between 10 to 5 years of blood collection, 20 CLL cases diagnosed within 5 years of blood collection), each bin was compared to 4,109 non-cases using Prentice-weighted Cox regression. For further details on the time-stratified analyses for CLL, see **Supplementary Figure 5**. C) Trajectory analysis of the hazard ratio of the top 10 proteins associated with DLBCL ranked by FDR-adjusted p-value. Cases were divided in 3 bins (45 DLBCL cases diagnosed over 10 years after blood collection, 21 DLBCL cases diagnosed between 10 to 5 years of blood collection, 14 DLBCL cases diagnosed within 5 years of blood collection), each bin was compared to 4,111 non-cases using Prentice-weighted Cox regression. For further details on the time-stratified analyses for DLBCL, see **Supplementary Figure 6**. D) Trajectory analysis of the hazard ratio of the top 10 proteins associated with MM ranked by FDR-adjusted p-value. Cases were divided in 3 bins (53 MM cases diagnosed over 10 years after blood collection, 33 MM cases diagnosed between 10 to 5 years of blood collection, 30 MM cases diagnosed within 5 years of blood collection), each bin was compared to 4,111 non-cases using Prentice-weighted Cox regression. For further details on the time-stratified analyses for MM, see **Supplementary Figure 8**.

We additionally performed separate analyses for the most common B-cell malignancy subtypes: CLL (n = 80), DLBCL (n = 80), FL (n = 51) and MM (n = 116) (**Supplementary Table 2**). We observed significant heterogeneity among the top 10 associated proteins for each subtype (Fig. 2A). While some proteins such as sCD23, CD28, CD72 and FCRL3 were associated across multiple B-cell malignancy subtypes, others such as sBCMA, SLAMF7 and IGSF3 were specifically associated with a particular B-cell malignancy subtype. Strikingly, all the top proteins associated with B-cell lymphoma development (sCD23, FCMR, FCRL1, FCRL3, SELL, SEMA7A and SEMA4A) were most strongly associated with CLL development, suggesting CLL was a major driver of the top protein associations observed in the grouped analysis.

### CLL

We observed 589 protein-CLL associations (617 aptamers), of which 544 were associated with an increased risk and 45 were associated with a decreased CLL risk (**Supplementary Fig. 4A, Supplementary Table 2**). CLL development was associated with enrichment of proteins associated with the spliceosome, ribosome, base excision repair, viral carcinogenesis, p53 signaling and the polycomb repressive complex (**Supplementary Fig. 4D, Supplementary Fig. 5D**). Time-stratified analyses revealed an exponential increase in the number of FDR-significant CLL-protein associations among participants with a blood draw collected less than 5 years prior to diagnosis (1094 proteins), compared to CLL cases with a blood draw collected between 5 to 10 years prior to diagnosis (41 proteins) and CLL cases with a blood draw collected between over 10 years prior to diagnosis (6 proteins, **Supplementary Fig. 5ABC**). The only protein significantly associated across all time intervals from > 10 years to < 5 years before CLL diagnosis was FCMR (**Supplementary Fig. 5E**). Trajectory analyses for the top 10 proteins associated with CLL ranked by FDR-adjusted P-value revealed increasing hazard ratios for FCMR, sCD23, SELL and IGSF3 in blood samples drawn within 5 to 10 years before diagnosis, compared to participants with blood samples drawn over 10 years before diagnosis (Fig. 2B). The hazard ratio for FCMR, FCRL3, SLAMF6, CD72, SEMA4A, CLSTN1, SIGLEC6 increased sharply among participants with a blood sample drawn within 5 years before diagnosis.

### DLBCL

We observed 34 protein-DLBCL associations (36 aptamers), of which 33 were associated with an increased risk and 1 was associated with a decreased DLBCL risk (**Supplementary Fig. 4B, Supplementary Table 2**). DLBCL development was associated with PD-1 signaling, T-cell receptor signaling, homologous recombination and antigen processing and presentation (**Supplementary Fig. 4E**). Time-stratified analyses revealed an increase in the number of FDR-significant DLBCL-protein associations among participants with a blood draw collected less than 5 years prior to diagnosis (48 proteins), compared to DLBCL cases with a blood draw collected between 5 to 10 years prior to diagnosis (18 proteins) and DLBCL cases with a blood draw collected between over 10 years prior to diagnosis (11 proteins, **Supplementary Fig. 6ABC**). No proteins were consistently associated across all time intervals from > 10 years to < 5 years before DLBCL diagnosis (**Supplementary Fig. 6E**). Trajectory analyses for the top 10 proteins associated with DLBCL ranked by FDR-adjusted P-value revealed relatively stable hazard ratios for all proteins until 5 years before DLCBL diagnosis (Fig. 2C). Among individuals with a blood draw less than 5 years before DLBCL diagnosis, we observed an increasing hazard ratio for sCD23, LAG3, FCRL1 and CD28.

### FL

We observed 20 protein-FL associations (21 aptamers), of which 18 were associated with an increased risk and 2 were associated with a decreased FL risk (**Supplementary Fig. 4C, Supplementary Table 2**). FL development was associated with an enrichment of proteins associated with the hematopoietic cell lineage, T cell receptor signaling pathway and the phospholipase D signaling pathway (**Supplementary Fig. 4F**). No time-stratified analyses were performed for FL due to the limited sample size for this subgroup (n = 51).

### Overlap between B-cell lymphoma subtypes

Overlap of the protein associations with CLL, DLBCL and FL was limited to sCD23, CD28, FCRL3 and CD72 (Fig. 2A, **Supplementary Fig. 4G**). All 3 entities were associated with BCR signaling and cytokine signaling (**Supplementary Fig. 4DEF, Supplementary Table 4**). Interestingly, the overlap between DLBCL and FL included several proteins involved in germinal center signaling, namely: CXCL13, follicular dendritic cell-secreted protein (FDCSP) and CCL21 (**Supplementary Fig. 4G)**.

### MM

We observed associations prior to MM diagnosis (n = 116) for 30 unique proteins (32 aptamers), of which 26 were associated with an increased risk and 4 were associated with a decreased MM risk (**Supplementary Fig. 7A, Supplementary Table 2**). The top hits included plasma cell activation markers previously associated with MM, such as sBCMA (TNFRSF17), SLAMF7, TACI (TNFRSF13B) and BAFF (TNFSF13B, inverse association).^32–36^ We also observed associations for several proteins that have only very recently been linked to MM development, namely contactin-5 (CNTN5), IL5RA and QPCT (Fig. 2A and 2D).^29,30^ Pathway analysis revealed an enrichment of proteins associated with antibody production and plasma cell activation, JAK-STAT signaling and cytokine signaling in the years prior to MM diagnosis (**Supplementary Fig. 7B**). Overlap between protein associations with MM and BCL was limited to 8 proteins (**Supplementary Fig. 7D**). Time-stratified analyses revealed an increase in the number of FDR-significant MM-protein associations among participants with a blood draw collected less than 5 years prior to diagnosis (95 proteins), compared to MM cases with a blood draw collected between 5 to 10 years prior to diagnosis (14 proteins) and MM cases with a blood draw collected between over 10 years prior to diagnosis (7 proteins, **Supplementary Fig. 8, Supplementary Table 3**). sBCMA, CNTN5, IL5RA and SLAMF7 were consistently associated across all time intervals from > 10 years to < 5 years before MM diagnosis (**Supplementary Fig. 8E**). Trajectory analyses for the top 10 proteins associated with MM ranked by FDR-adjusted P-value revealed a linear increase in hazard ratio over time for sBCMA (Fig. 2D). We additionally observed an increasing hazard ratio for CD48, QPCT, SLAMF7 and LY9 among participants with a blood draw less than 5 years before MM diagnosis.

### Germinal center-derived B-cell lymphoma and non-germinal center-derived B-cell lymphoma

**Figure 3:**
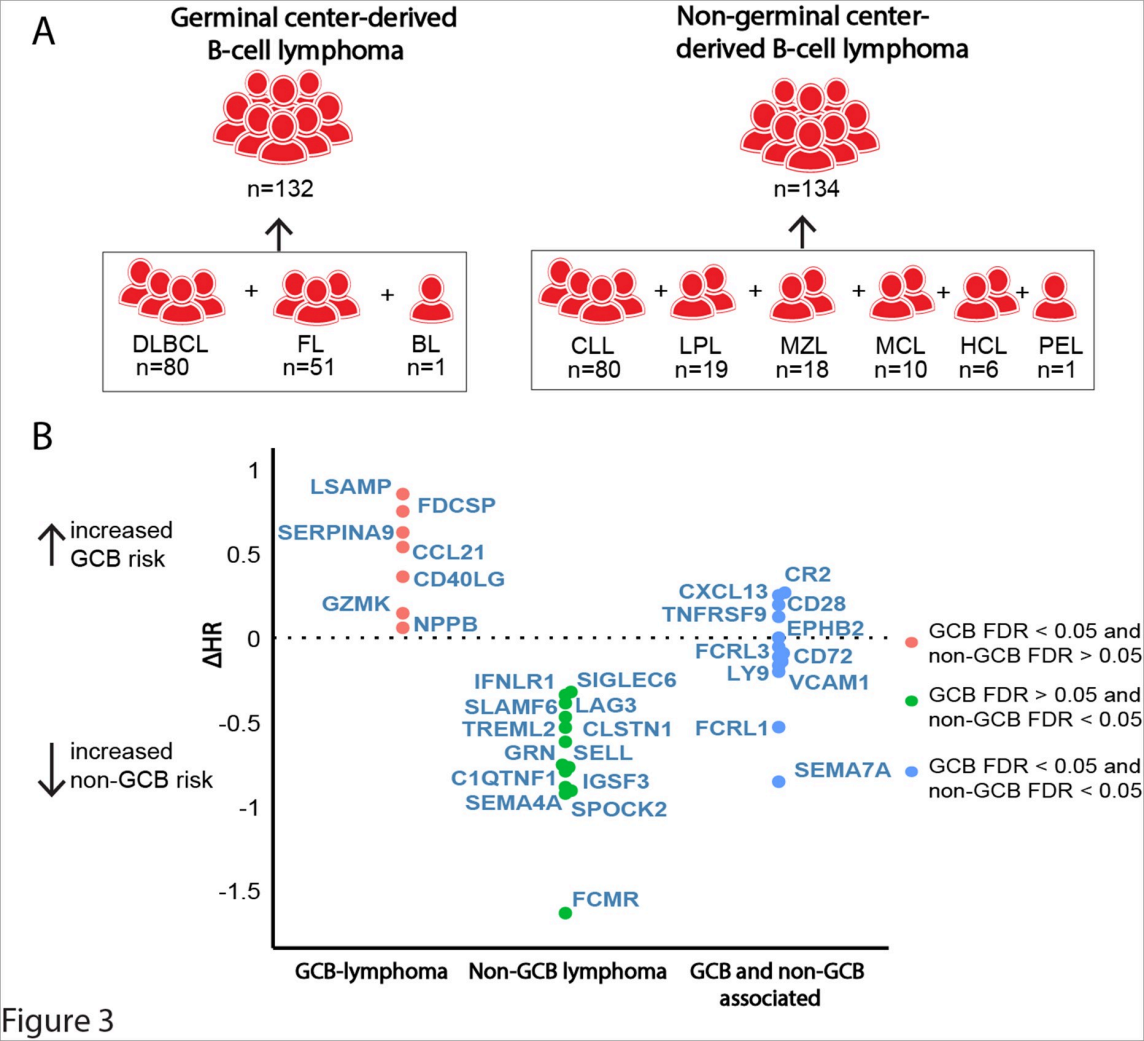
Protein associations with germinal center-derived B-cell lymphoma and non-germinal center-derived B-cell lymphoma. A) Overview of grouping for germinal center-derived B-cell lymphoma and non-germinal center derived B-cell lymphoma groups. Prentice-weighted Cox regression models for the risk of GCB-lymphoma included 4,106 non-cases and the 132 germinal center-derived B-cell lymphoma cases. Prentice-weighted Cox regression models for the risk of non-GCB lymphoma included 4,101 non-cases and the 134 non-germinal center-derived B-cell lymphoma cases. B) Comparison of the change in hazard ratio between the models for germinal center-derived B-cell lymphoma risk and non-germinal center-derived B-cell lymphoma risk. Proteins were divided into 3 groups, only FDR-significantly associated with germinal center-derived B-cell lymphoma risk (red), only FDR-significantly associated with non-germinal center derived B-cell lymphoma risk (green) or FDR-significantly associated with both groups (blue). The top 20 proteins (ranked by FDR-adjusted P-value) associated with germinal center-derived B-cell lymphoma risk and the top 20 proteins associated with non- germinal center-derived B-cell lymphoma risk were included in the figure. For further details on the analyses relating to germinal center B-cell lymphoma and non-germinal center B-cell lymphoma see **Supplementary Figure 9 and 10**.

Based on the results of our initial analyses, we hypothesized that the germinal center reaction may be one of the dominant driving mechanisms behind the observed protein-BCL associations. To further investigate this hypothesis, we performed separate analyses for germinal center-derived B-cell lymphoma (DLBCL, FL and Burkitt lymphoma, n = 132) and non-germinal center-derived B-cell lymphoma cases (CLL, mantle cell lymphoma, lymphoplasmacytic lymphoma, marginal zone lymphoma, hairy cell leukemia and primary effusion lymphoma, n = 134, Fig. 3A, **Supplementary Table 1**).^37^

In our fully-adjusted models, we observed 28 unique proteins associated with an increased risk of germinal center-derived B-cell lymphoma (**Supplementary Fig. 9AB, Supplementary Table 2**). Top proteins specifically associated with germinal center-derived B-cell lymphoma risk included LSAMP, FDCSP, SERPINA9, CCL21 and CD40LG (Fig. 3B). Among germinal center-derived B-cell lymphomas, only FDCSP showed consistent associations across all pre-diagnostic time intervals (**Supplementary Fig. 10ABCE**).

In contrast, non-germinal center-derived B-cell lymphoma development was associated with 187 unique protein associations, of which 175 were associated with an increased risk and 13 were associated with a decreased risk of non-germinal center-derived B-cell lymphoma (**Supplementary Fig. 9CD, Supplementary Table 2**). Top proteins specifically associated with non-germinal center-derived B-cell lymphoma included FCMR, SPOCK2, IGSF3 and SEMA4A (Fig. 3C). Time-stratified analyses for non-germinal center-derived B-cell lymphoma revealed 4 proteins (FCMR, FCRL3, SEMA4A and SLAMF6) associated with non-germinal center-derived B-cell lymphoma from early development (> 10 years before diagnosis) until less than 5 years before diagnosis (**Supplementary Fig. 10FGHJ**).

### Subtype analysis for Hodgkin lymphoma and T-cell lymphoma

Due to the rarity of T-cell lymphoma (TCL, n = 15) and HL cases (n = 23) in our cohort, statistical power was very limited for these sub-analyses. We observed 26 protein-HL associations (**Supplementary Fig. 7CD**). Overlap between protein associations with HL and BCL was limited to 4 proteins (CD28, CXCL13, FAM20B and IL-18BP, **Supplementary Fig. 7D**). We observed 49 unique protein-TCL associations (**Supplementary Fig. 11A, Supplementary Table 2**). Pathway analysis revealed an enrichment of proteins associated in processes such as cell adhesion, growth hormone, cell cycle, transcriptional dysregulation and the p53 signaling pathway (**Supplementary Fig. 11B, Supplementary Table 4**). Notably, only two proteins (CD14 and IGF1) overlapped between BCL and TCL, suggesting largely distinct proteomic risk profiles (**Supplementary Fig. 11C)**.

### Cross-cohort comparison with ARIC and the UK Biobank

**Figure 4:**
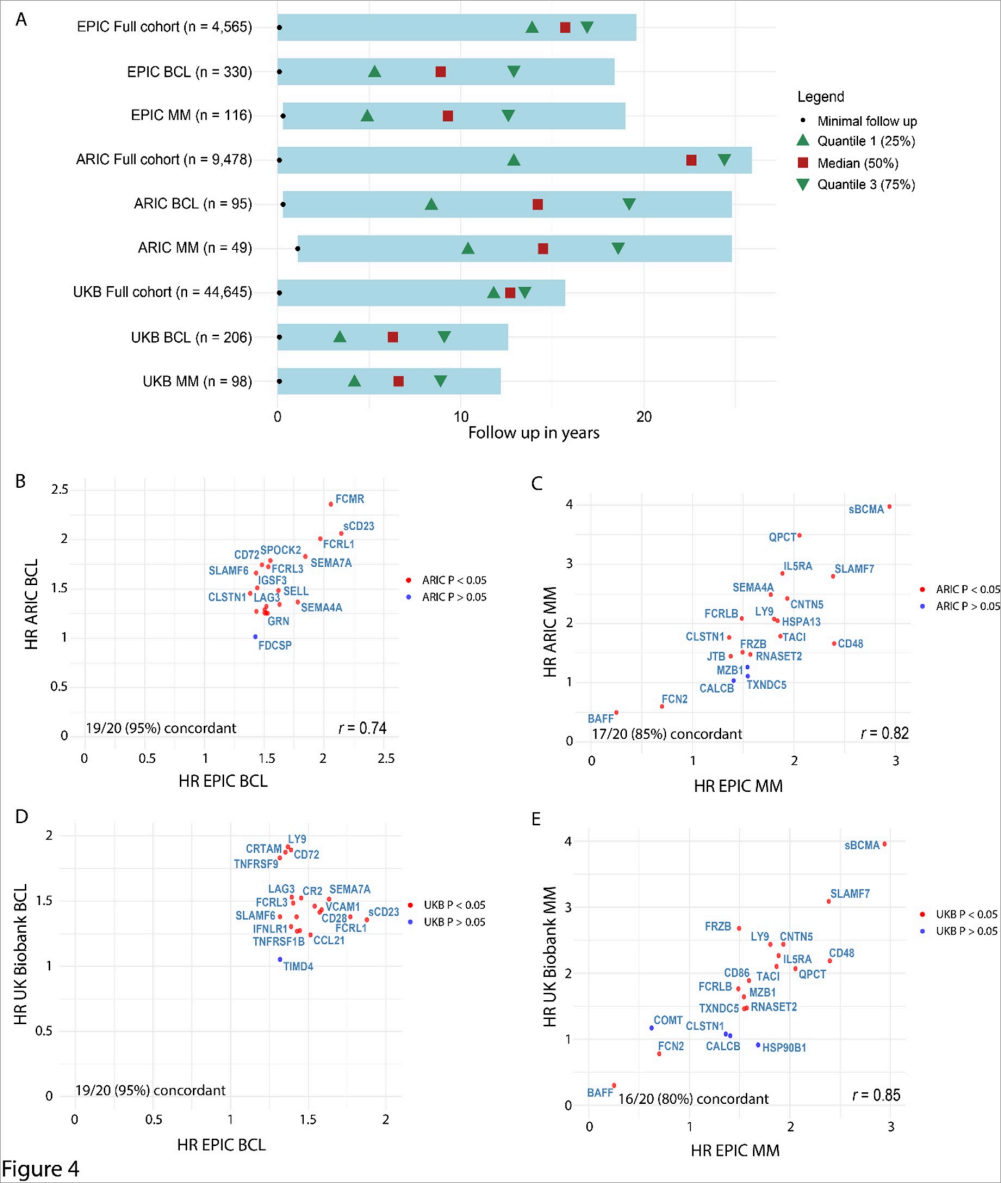
Cross-cohort comparison of top 20 overlapping proteins ranked by FDR-adjusted P-value with the UK Biobank (Olink) and ARIC (SomaScan) cohorts. A) Schematic overview of follow up time in the EPIC, ARIC and UK-biobank cohorts. Each bar covers the entire follow up range in each group. Minimal follow up time is indicated with a black dot, median follow up time with a red square and the 1^st^ and 3^rd^ quantiles are indicated with green triangles. B) Hazard ratio of top 20 FDR-significant protein-associations in EPIC plotted against the hazard ratios observed for these proteins in ARIC for B-cell lymphoma (including CLL). Correlation coefficient in bottom right corner indicates the significant positive correlation found for the hazard ratios across the studies. Each dot represents a single protein. Red dots reached FDR significance in EPIC and nominal significance in either UK Biobank or ARIC. Blue dots are significant only in EPIC. C) Hazard ratio of top 20 FDR-significant protein-associations in EPIC plotted against the hazard ratios observed for these proteins in ARIC for MM. Correlation coefficient in bottom right corner indicates the significant positive correlation found for the hazard ratios across the studies. D) Hazard ratio of top 20 FDR-significant protein-associations in EPIC plotted against the hazard ratios observed for these proteins in the UK biobank for BCL (not including CLL). No significant correlation was observed between the hazard ratios. E) Hazard ratio of top 20 FDR-significant protein-associations in EPIC plotted against the hazard ratio’s observed for these proteins in the UK Biobank for MM. Pearson correlation coefficient in bottom right corner indicates the significant positive correlation found for the hazard ratios across the studies.

We compared our top 20 protein-cancer associations by FDR-adjusted P-value within EPIC with nominal protein-cancer associations observed in the ARIC and UK Biobank cohorts (Fig. 4). The ARIC cohort had the longest follow up, but a smaller sample size in terms of included cases (Fig. 4A). The UK-biobank cohort had the largest overall sample size and the shortest follow up (Fig. 4A). The EPIC cohort included the largest number of included lymphoid malignancy cases and had an intermediate follow up length compared to the other two cohorts (Fig. 4A). The EPIC (6,412 proteins) and ARIC cohorts (4,712 proteins) were measured using SomaScan technology, while the UK biobank cohort (3,072 proteins) was measured using the Olink Explore assay.

The top 20 FDR significant proteins within EPIC showed high concordance in ARIC for BCL (95%, HR *r* = 0.74), CLL (90%, HR *r* = 0.68) and MM (85%, HR *r* = 0.82) (Fig. 4BC, **Supplementary Fig. 12A, Supplementary Table 5**). The top 20 FDR significant proteins within EPIC showed high concordance in the UK Biobank in terms of nominal significance for BCL (95%, *r* = n.s.), DLBCL (70%, *r* = n.s.) and MM (80%, HR *r* = 0.85) (Fig. 4DE, **Supplementary Fig. 12B, Supplementary Table 5)**. However, no significant correlation was observed in size of the HR between EPIC and UK Biobank for the top 20 overlapping proteins associated with BCL and DLBCL (Fig. 4DE). Top proteins that were consistently associated with B-cell lymphoma across the EPIC, ARIC and UK Biobank cohorts included: CXCL13, sCD23, FCRL1, FCRL3, SEMA7A, CD72, CD48, LY9 and VCAM1 (Table 2, **Supplementary Table 5)**. Top proteins consistently associated with MM across the EPIC, ARIC and UK Biobank cohorts included: sBCMA, CNTN5, IL5RA, CD48, SLAMF7, TACI, BAFF, QPCT, FCRLB and LY9 (Table 2, **Supplementary Table 5)**.

## Discussion

In this large-scale prospective study evaluating 6,412 circulating proteins in 4,565 EPIC participants, we identified key proteomic markers linked to early lymphoid malignancy pathogenesis (*e.g*. sBCMA, CXCL13, sCD23, CD28, CD72, FCRL1, FCRL3, SEMA4A, and SEMA7A) and reveal several promising novel protein markers associated specifically with germinal center-derived B-cell lymphoma (*e.g*. FDCSP, CCL21, CD40LG and SERPINA9) and non-germinal center-derived B-cell lymphoma subtypes (*e.g*. FCMR and SELL). Validation in the UK Biobank and ARIC cohorts revealed high concordance among the top 20 FDR-significant proteins. Our observations extend previously published data describing elevated levels of B-cell activation markers and cytokines prior to lymphoma diagnosis.^21,22,25,28,33,38^

Predictably, not all of the identified prediagnostic protein-lymphoma associations were novel. In particular, sCD23 serum levels have been previously linked to early development of BCL, especially of CLL.^27^ CXCL13 serum levels have been previously linked to DLBCL development.^27^ sBCMA and SLAMF7 plasma levels have been previously linked to MGUS and progression to MM.^32,33,36^ Additionally, some of the other identified candidate proteins overlap with markers of active disease. A recent study in the Uppsala-Umeå Comprehensive Cancer Consortium (U-CAN) biobank measured 1463 proteins in treatment-naïve diagnostic plasma samples from 48 CLL, 55 DLBCL and 38 MM patients using Olink Explore PEA technology.^29^ Notable proteins associated with active disease included SLAMF7, MZB1, QPCT, BAFF and CNTN5 for MM, FCRL1, FCRL3, IGSF3, SEMA7A, SIGLEC6, TCL1A and sCD23 for CLL and SERPINA9 and CXCL13 for DLBCL. Similarly, FCMR and SELL levels are increased in the plasma of CLL patients.^39,40^ CD48 expression is upregulated in MGUS and MM cells and soluble CD48 is increased in the serum of patients with leukemia or lymphoma.^41,42^ LY9 (CD229) serum levels are increased in MM patients at diagnosis.^43^ Key novel prediagnostic plasma markers for lymphoid malignancy development identified in our current study include: CD72, CD28 and SLAMF6 for CLL, FDCSP, CD72, CD28, LAG3 and INSL4 for DLBCL, FDCSP, CD72 and CD28 for FL, TACI and FCRLB for MM. Notably, several of these markers have previously been described to be highly expressed on the cell surface of the malignant cells.^44–46^ These findings indicate that there is considerable overlap between key prediagnostic lymphoid malignancy markers and markers of overt disease, suggesting that early disease biology may be driven by similar molecular pathways as clinically apparent malignancy.

Growth and survival of malignant B cells is sustained through constitutive activation of normal B cell signaling pathways.^15,47^ Malignant B-cells exploit these pathways through a combination of gain-of-function mutations that activate downstream signaling mediators, loss-of-function mutations that inactivate negative signaling regulators and autocrine receptor activation. Our study revealed an enrichment of circulating proteins associated with several of these B-cell signaling pathways, including BCR signaling, cytokine signaling, epigenetic dysregulation, JAK kinase pathways and NF-κB activation.^15^ Increased plasma levels of proteins associated with BCR signaling are consistent with the central role of the BCR as a survival signal during lymphomagenesis, both in response to various (auto)antigens and through antigen-independent signaling following crosslinking of the BCR on the cell surface.^48,49^ Similarly, constitutive NF-κB activation results in continuous lymphocyte proliferation and survival, a critical pathogenic factor in lymphoma.^47^ The top pathway associated with BCL development involved altered cytokine signaling due to viral protein interaction, consistent with the essential role of viruses such as Epstein-Barr virus in the pathogenesis of many BCL subtypes.^50,51^ Germinal center signaling through CD40L, CCL21, FDCSP and CXCL13 appeared to profoundly shape the plasma proteome of germinal center-derived compared to non-germinal center-derived B-cell lymphoma subtypes.^52–56^

Our pathway enrichment analysis highlights the biological heterogeneity between lymphoid malignancy subtypes, each showing subtly distinct plasma proteomic profiles. For example, the proteomic profile observed prior to MM diagnosis was strongly distinct from the other lymphoid malignancy subtypes, with the top proteins reflecting the altered survival signals that plasma cells require compared to other B-cell subtypes (e.g. sBCMA, SLAMF7, TACI and BAFF). In contrast, the proteomic profile associated with CLL development was marked by increased levels associated with the spliceosome, base excision repair and NF-kB signaling and particular proteins such as FCMR, SELL and IGSF3. Moreover, the number of protein associations varied across subtypes, with more localized subtypes such as FL and DLBCL yielding significantly fewer protein associations than leukemic forms, like CLL. Nonetheless, shared features were observed across subtypes, notably involving cytokine and BCR signaling pathways. These observations suggest that lymphoid malignancies are not a homogeneous group but should be considered as distinct entities, each with its own molecular pathogenesis.

Cross-cohort comparison of our results with the ARIC study (SomaScan) and the UK Biobank (Olink) revealed concordant associations for 70%−95% of the top 20 proteins identified in EPIC ranked by FDR significance, indicating strong agreement between the cohorts for these top hits. Furthermore, we observed a positive correlation in the observed HRs for the proteins in ARIC and EPIC. In the UK Biobank, we only observed a significant correlation in the HRs for the top 20 proteins associated with MM, not for BCL and DLBCL. Observed discrepancies for certain proteins may reflect biological heterogeneity, differences in subtype distribution and follow up duration between the cohorts, or technical variation across proteomic platforms. Indeed, previous studies comparing the Olink and SomaScan platforms observed only a moderate positive correlation for overlapping proteins (*r* = 0.3–0.4).^57–61^ Protein families consistently associated with lymphoid malignancy development across all three cohorts included members of the Fc-receptor family, the semaphorins and the TNF superfamily.^30^

Importantly, the extended indolent period prior to lymphoid malignancy blurs the distinction between early risk markers and indicators of subclinical disease.^30^ It currently remains unclear at what stage irreversible malignant conversion occurs.^13^ Even risk markers detected over a decade prior to lymphoid malignancy diagnosis may still be attributed to reverse causality. One potential strategy to evaluate the impact of reverse causality would be to pair proteomic plasma measurements with an early cancer detection test, *e.g*. cell-free (cf)DNA methylation, to identify occult malignant populations.^62–64^ This approach would allow for the enhanced differentiation between those individuals who are still in a premalignant inflammatory state and those individuals with occult malignant disease, facilitating preventative intervention. However, cfDNA-based early detection tests generally have lower sensitivity for indolent and very early-stage cancers, as these tumors shed relatively little cfDNA into circulation.^64^ Therefore, the utility of such tests may be limited for certain lymphoid malignancy subtypes.

The clinical pathway for applying the identified biomarkers remains to be defined. For MM, clinical utility could consist of early treatment with Lenalidomide at the high risk smouldering MM stage, which has been shown to improve outcomes for MM patients.^65,66^ Similar results might be potentially achieved in the future for aggressive lymphoma subtypes, such as DLBCL and MCL. For other, more indolent lymphoid malignancy subtypes, the path to clinical utility is more challenging. Early identification of individuals with CLL and FL at increased risk of transformation to a more aggressive B-cell lymphoma may provide additional opportunities for early therapeutic intervention.^67,68^ However, the significant side-effects of chemotherapy and targeted therapeutic options make them an unattractive treatment option for early stage disease.

Ideally, prevention strategies would rely on interventions with a more favorable risk–benefit profile. These could include targeting chronic inflammation, which is thought to play a key role in lymphoma pathogenesis,^69^ reducing environmental exposures associated with increased risk (*e.g*. benzene, pesticides, viral infections),^70–73^ and promoting protective lifestyle changes such as improvements in diet and physical activity.^74–78^ Such public health interventions have the added advantage of benefiting multiple disease areas, further strengthening their risk–benefit profile. Ultimately, detection of premalignant inflammatory states and occult disease is a crucial first step towards the development of effective and safe prevention and early intervention strategies.

Strengths of the current study include its large sample size, the long follow-up period prior to disease onset, the large quantity and breadth of unique proteins measured, the cross-cohort validation of top associations across cohorts, and the ability to evaluate marker associations across the major lymphoma subtypes. Limitations of our current study include a lack of detailed data on clinical characteristics and disease outcome after lymphoid malignancy diagnosis and the absence of repeated samples to study individual trajectories. A limitation in our analyses studying germinal center-derived B-cell lymphoma is that we did not have sufficiently detailed descriptive data available to divide the DLBCL cases in Germinal Center B-Cell-Like DLBCL and Activated B-Cell-Like DLBCL. Potentially, the observed associations could be strengthened by enhanced stratification based on B-cell biology.

Our findings highlight several processes relevant to the early stages of lymphoid malignancy development, including epigenetic alterations, Fc-receptor signaling, BCR signaling, and germinal center signaling. Understanding which cells produce the proteins we detect in the circulation will require single-cell technology, ideally supplemented by spatial transcriptomics to relate our observations back to tissue architecture. For example, research into HL has implicated an essential role for rosetting (i.e clusters of surrounding) CD4^+^ T-cells in Hodgkin tumor cell survival.^79^ These rosetting T-cells express CXCL13, CD28 and TNFRSF18, proteins that were elevated in plasma years prior to HL diagnosis in the current study.^80,81^ Additionally, longitudinal dynamics of early disease markers may hold potential to capture key transitions during early disease development that predict progression to overt lymphoma and aid in clinical risk stratification.

The predominance of cell surface proteins among those associated with lymphoid malignancy risk raises questions about whether these proteins are actively secreted or reflect shedding from malignant or pre-malignant cells. While the merit of a protein risk factor is not necessarily affected by its origin, understanding the mechanism by which circulating protein levels are altered would aid interpretation of fluctuations in protein levels over time to lymphoid malignancy diagnosis.

In summary, our study provides new insights into the molecular mechanisms underlying lymphoid malignancy. The proteins identified, many of which are cell surface markers, represent promising targets for biomarker-driven strategies for early detection, interception and prevention. Additionally, these proteins may provide crucial insights into lymphoma etiology. Future research leveraging multi-omics approaches, single-cell analyses, and spatial transcriptomics will be critical to fully unravel the origins and progression of these diseases. By bridging early molecular alterations to overt malignancy, these findings lay the groundwork for earlier detection and more precise intervention strategies in lymphoid cancers.

## Online Methods

### EPIC participants

EPIC is a prospective cohort study of approximately 521,000 participants (aged 35–70 years) recruited between 1992 and 2000 in 23 centers located in 10 European countries.^82^ Among the participants, ~ 70% were women and blood samples were collected from ~ 75%. All participants provided informed consent. In EPIC, incident first primary cancer cases (excluding non-melanoma skin cancers) were identified through a combination of center-specific methods, including health insurance records, cancer and pathology registries and active follow-up through study participants and their next-of-kin. Follow-up for each participant and event of interest began upon inclusion in the study and ended upon the occurrence of the event, loss to follow-up, or the last date of ascertainment, whichever came first. Cancer endpoints were defined as the first incident cancer diagnosis, coded using the 10th revision of the WHO’s International Statistical Classification of Diseases (ICD-10)]. In the current study, blood samples from a total of 4,565 individuals recruited in the UK, the Netherlands, Spain, and Italy underwent proteomic analysis by Somalogic using the SomaScan 7k Assay. The EPIC study was conducted in accordance with the Declaration of Helsinki. The study was approved by the local ethical committees in participating countries and the International Agency for Research on Cancer (IARC) ethical committee (EPIC25–34). All participants provided written informed consent for data collection and storage, as well as individual follow-up before study entry. Approval for the ARIC study was received from the Institutional Review Board at each study center (Johns Hopkins Medicine IRB-3, IRB00311861). All participants provided informed consent.

### Study design

For the current study, we used a case-cohort study design (Fig. 1A). We initially included 449 lymphoid malignancy cases from the total EPIC cohort (~ 500,000 individuals) in the study. For the controls, we selected a random subcohort (n = 4,116) from the total EPIC cohort (~ 500,000 individuals). Selection into the sub-cohort was conducted without selecting on future case status, meaning that some participants (n = 35) diagnosed with a lymphoid malignancy subsequently were included by chance. The subcohort consists of 3,715 cancer-free individuals, 366 individuals with a solid cancer diagnosis (*e.g*. breast or colorectal cancer) and 35 individuals with a lymphoid malignancy diagnosis (**Supplementary Fig. 1A**). Therefore, we included a total of 484 lymphoid malignancy cases and 4,081 non-cases (Fig. 1A, **Supplementary Fig. 1B**).

In each of the analyses described below, we included the lymphoid malignancy subset of interest and compared them to the full sub-cohort through Prentice-weighted Cox regression. We analyzed BCL (n = 330), TCL (n = 15) and MM (n = 116) separately, as MM is a disease of the plasma cells, a strongly differentiated B-cell subset. Additionally, subtype-specific analyses were performed for CLL (n = 80), DLBCL (n = 80) and FL (n = 51), the three most prevalent subtypes of BCL, as well as for Hodgkin lymphoma (HL, n = 23). We then divided the BCL cases in germinal center B-cell lymphoma (encompassing DLBCL, FL and Burkitt lymphoma, n = 132) and non-germinal center B-cell lymphoma cases (encompassing CLL, mantle cell lymphoma, lymphoplasmacytic lymphoma, marginal zone lymphoma, hairy cell leukemia and primary effusion lymphoma, n = 134) and ran separate analyses for each group (Fig. 3B). A graphical overview of the analytical strategy is shown in **Supplementary Fig. 1B** and a full overview of the lymphoid malignancy cases by subtype and by group is provided in **Supplementary Table 1**.

### Proteomic measurement, processing and quality control

Plasma samples from all 4,565 participants underwent high throughput proteomic profiling by Somalogic using the SomaScan 7k assay. Briefly, the SomaScan platform utilizes modified nucleotides (Slow Off-rate Modified Aptamers), which bind directly to specific proteins. The aptamers are tagged with a fluorophore, allowing for quantification in relative fluorescent units (RFUs) using a DNA microarray.^83^ For some targets, the SomaScan 7k panel includes aptamers that bind to isoforms of the same protein or can bind to the same protein at different sites, extending the detectable range of proteoforms. The SomaScan 7k Assay uses 7,596 aptamers to measure 6,432 proteins (UniProt IDs).

In our main analysis, we used RFUs normalized by Somalogic through the following steps: hybridization normalization, intraplate median normalization, plate scaling and calibration, and adaptive normalization to a population reference. The normalized RFUs were then log_10_-transformed to reduce skewness. Samples detected as outliers using PCA and a local outlier factor using a Tukey rule modified to account for skewness and multiple testing were excluded.^84,85^ We corrected the measurements for each aptamer for plate effects estimated in linear mixed effect models adjusted for center, age, sex, BMI, smoking status, and incidence of cancer, to preserve possible biological variation due to these factors.^86^ Finally, measurements of each aptamer were centered and scaled so that their mean and standard deviation were respectively 0 and 1 in the sub-cohort. Log-transformed relative abundance of each aptamer was capped at greater or less than 5 standard deviations from the mean. Original deidentified data is available through the International Agency for Research on Cancer’s Scientific IT Platform. Interested parties may contact epic@iarc.who.int.

### Statistical analysis

We estimated hazard ratios (HRs) and 95% confidence intervals (CI) for the major lymphoid malignancy subsets separately using Prentice-weighted Cox regression models as appropriate for analyzing case-cohort data with age as the underlying time variable. The minimally adjusted models were stratified by age group at recruitment (5-year categories), the center at which the blood sample was collected, and sex. Multivariable-adjusted models were additionally adjusted for smoking status and BMI, two known risk factors for lymphoid malignancy subtypes. Changes in hazard ratio in the fully adjusted vs the minimally adjusted model are shown in **Supplementary Fig. 2**. A Benjamin-Hochberg false discovery rate (FDR) control was applied for multiple testing (FDR-adjusted P < 0.05). Time-stratified analyses were employed for lymphoid malignancy subtypes that included at least 80 cases, meaning BCL, MM, CLL and DLBCL, to explore dynamics in protein-lymphoid malignancy associations over time to lymphoid malignancy diagnosis. For the time-stratified analyses, we divided the cohort into bins based on time to diagnosis (> 10 years, 5–10 years and < 5 years) prior to running Prentice-weighted Cox regression models. All correlation coefficients were determined using Pearson correlation. All analyses were conducted using R version 4.1.2.

### Pathway Enrichment analysis

To identify whether prospectively associated proteins were associated with specific molecular pathways we performed gene-set enrichment analysis (GSEA) for all proteins identified in the Cox proportional hazards model with an FDR-adjusted P-value < 0.05. GSEA was performed using an active-subnetwork-oriented approach, implemented with pathfindR (version 2.4.1), which maps each gene onto a protein-protein interaction network to better account for gene interaction information, with each subnetwork identified using a greedy algorithm with 10 iterations.^87^ We used the BioGrid interaction database (version 4.4.232) for our protein-protein interaction network as it had the largest overlap with the protein-coding genes mapped to our aptamers and used all 580 currently available KEGG Pathway Database gene-sets for the pathway enrichment analysis. All significantly enriched KEGG pathways (P < 0.05) were included in supplementary table 4.

### Cross-cohort comparison with ARIC and UK Biobank

In the visit 2 of the Atherosclerosis Risk in Communities (ARIC) study, 4,712 plasma proteins were measured using the SomaScan 5K Assay (4,953 aptamers passing quality control) in 9,478 middle-aged adults (age 56.9 ± 5.7 years) at risk for a first primary cancer and who provided appropriate consent.^88,89^ Among them, 95 B-cell lymphoma (of which 40 were CLL) and 49 MM first primary cases were ascertained primarily by state cancer registry linkage over a maximum follow up of 25 years (Fig. 4A).^90,91^ We evaluated concordance of the nominal significance within ARIC for the top 20 FDR significant hits within EPIC for B-cell lymphoma, CLL and MM. Cox proportional hazards regression models were harmonized with EPIC and adjusted for age, race/field center, sex, smoking status, and BMI. In a recent UK Biobank study, 3072 proteins were measured in plasma in 54,306 middle-aged adults using Olink technology, including 206 lymphoma (of which 89 DLBCL), 130 leukemia (C91-C95) and 96 MM cases.^30^ We evaluated concordance of the nominal significance within the UK Biobank for the top 20 FDR significant hits within EPIC for total lymphoma (excluding CLL), MM and DLCBL. In the UK Biobank analysis, CLL was grouped under leukemia (including myeloid leukemias) rather than BCL, preventing a direct comparison.

## Supplementary Material

Supplementary Files

This is a list of supplementary files associated with this preprint. Click to download.

- 20250811Supplementalmaterial.pdf
- Supplementarytable2.xlsx
- 20250811Supplementarytables34and5.xlsx

## Figures and Tables

**Table 1:** Descriptive table. “Years blood draw to diagnosis” refers to the number of years between the moment of blood draw and lymphoid malignancy diagnosis. BMI and age are listed at recruitment.

	Total cases	BCL	MM	HL	TCL	Sub-cohort
	N = 484	N = 330	N = 116	N = 23	N = 15	N = 4,116
**BMI, mean (Q1–Q3)**	26.9 (23.8–29.1)	26.8 (23.8–28.9)	27.2 (24.2–29.8)	26.2 (25.5–28.11)	26.5 (23.5–28.9)	26.9 (23.9–29.5)
**Age in years, mean (Q1–Q3)**	55.4 (49.6–61.6)	55.5 (49.8–62.0)	55.1 (51.7–61.3)	49.1 (43.7–55.2)	55.5 (47.8–62.9)	51.4 (44.8–57.9)
**Years blood draw to diagnosis, mean (Q1–Q3)**	8.8 (5.2–12.7)	8.9 (5.3–12.9)	9.0 (4.9–12.6)	6.1 (2.5–8.4)	9.3 (5.8–13.5)	-
**Sex N (%)**						
Male	233 (48.2)	153 (46.4)	56 (48.3)	17 (74)	7 (46.7)	1573 (38.2)
Female	250 (51.8)	177 (53.6)	60 (51.7)	6 (26)	8 (53.3)	2543 (61.8)
**Smoking status N (%)**						
Non-smoker	235 (48.7)	168 (50.9)	53 (45.7)	6 (26.1)	8 (53.3)	2098 (51)
Former smoker	149 (30.8)	101 (30.6)	38 (32.8)	7 (30.4)	3 (20)	984 (23.9)
Current smoker	99 (20.5)	61 (18.5)	25 (21.5)	10 (43.5)	4 (26.7)	1034 (25.1)

Abbreviations: BCL, B-cell lymphoma; MM, multiple myeloma; HL, Hodgkin lymphoma; TCL, T-cell lymphoma;

BMI, body mass index; Q, quantile.

**Table 2:** Comparison of top protein-BCL and protein-MM associations across EPIC, ARIC and UK biobank cohorts. Table includes the top 10 proteins ranked by FDR-adjusted p-value in EPIC. Proteins that were not measured in all 3 cohorts were excluded. The 95% confidence interval of the HR is shown in brackets For all other significant protein associations in ARIC and UK biobank, see supplementary table 5.

Protein	Outcome	HR EPIC	FDR EPIC	HR ARIC	P-value ARIC	HR UK Biobank	P-value UK Biobank
sCD23	BCL	2.1 [1.9–2.4]	<0.0001	2.1 [1.7–2.6]	<0.0001	1.4 [1.1–1.6]	<0.001
FCRL1	BCL	2.0 [1.8–2.2]	<0.0001	2.0 [1.6–2.5]	<0.0001	1.4 [1.1–1.7]	<0.001
SEMA7A	BCL	1.8 [1.7–2.1]	<0.0001	1.8 [1.5–2.3]	<0.0001	1.5 [1.3–1.7]	<0.0001
CD48	BCL	1.6 [1.4–1.8]	<0.0001	1.4 [1.1–1.7]	<0.01	1.5 [1.2–1.8]	<0.0001
CXCL13	BCL	1.5 [1.4–1.6]	<0.0001	1.3 [1.0–1.6]	0.04	1.5 [1.4–1.6]	<0.0001
FCRL3	BCL	1.5 [1.4–1.7]	<0.0001	1.7 [1.4–2.1]	<0.0001	1.5 [1.3–1.8]	<0.0001
VCAM1	BCL	1.5 [1.4–1.7]	<0.0001	1.5 [1.2–1.9]	<0.001	1.4 [1.2–1.7]	<0.0001
LY9	BCL	1.5 [1.3–1.6]	<0.0001	1.8 [1.4–2.2]	<0.0001	1.9 [1.6–2.3]	<0.0001
CD72	BCL	1.5 [1.4–1.6]	<0.0001	1.7 [1.4–2.1]	<0.0001	1.9 [1.6–2.2]	<0.0001
CCL21	BCL	1.3 [1.2–1.5]	<0.0001	0.9 [0.8–1.2]	0.7	1.2 [1.1–1.4]	<0.01
sBCMA	MM	2.9 [2.5–3.5]	<0.0001	4.0 [2.9–5.5]	<0.0001	4.0 [3.4–4.6]	<0.0001
CNTN5	MM	1.9 [1.7–2.2]	<0.0001	2.4 [1.8–3.2]	<0.0001	2.4 [2.1–2.8]	<0.0001
IL5RA	MM	1.9 [1.6–2.2]	<0.0001	2.8 [2.1–3.8]	<0.0001	2.3 [2.0–2.5]	<0.0001
CD48	MM	2.4 [2.0–2.9]	<0.0001	1.7 [1.2–2.2]	<0.001	2.2 [1.9–2.5]	<0.0001
SLAMF7	MM	2.4 [2.0–2.8]	<0.0001	2.8 [2.1–3.8]	<0.0001	3.1 [2.7–3.5]	<0.0001
TACI	MM	1.9 [1.6–2.2]	<0.0001	1.8 [1.3–2.4]	<0.0001	2.1 [2.0–2.2]	<0.0001
BAFF	MM	0.3 [0.2–0.3]	<0.0001	0.5 [0.4–0.7]	<0.0001	0.3 [0.3–0.4]	<0.0001
QPCT	MM	2.1 [1.8–2.4]	<0.0001	3.5 [2.6–4.7]	<0.0001	2.1 [1.9–2.3]	<0.0001
FCRLB	MM	1.5 [1.3–1.7]	<0.0001	2.1 [1.6–2.7]	<0.0001	1.8 [1.7–1.9]	<0.0001
LY9	MM	1.8 [1.6–2.1]	<0.0001	2.1 [1.6–2.8]	<0.0001	2.4 [2.1–2.8]	<0.0001

Abbreviations: BCL, B-cell lymphoma; MM, multiple myeloma; HR, hazard ratio; FDR, false discovery rate.

## Data Availability

Original deidentified data is available through the International Agency for Research on Cancer’s Scientific IT Platform. Preprocessing and analytical scripts are available on request to allow the replication of findings by researchers with EPIC data access. Interested parties may contact epic@iarc.who.int or r.c.h.vermeulen@uu.nl. ARIC data, including the data used in this analysis, can be accessed by the following policy: https://sites.cscc.unc.edu/aric/sites/default/files/public/listings/ARIC%20data%20sharing.pdf. ARIC data also are available via BioLINCC (controlled access database). Researchers can apply to use the UK Biobank resource for health-related research that is in the public interest (https://www.ukbiobank.ac.uk/register-apply/). Further information is available from the corresponding author upon request. Source Data are provided with this paper.
